# Swine Xenografts Share Few Predicted Indirectly Recognisable SLA‐Derived Epitopes With HLA‐Derived Epitopes From Human Kidney Grafts

**DOI:** 10.1111/tan.70291

**Published:** 2025-06-21

**Authors:** Benedict M. Matern, Eric Spierings, Emma Peereboom, Matt Tector, Joseph Tector, Massimo Mangiola, Robert A. Montgomery, Matthias Niemann

**Affiliations:** ^1^ PIRCHE AG Berlin Germany; ^2^ University Medical Center Utrecht Utrecht the Netherlands; ^3^ Makana Therapeutics Miami Florida USA; ^4^ University of Miami Miami Florida USA; ^5^ NYU Langone Transplant Institute New York New York USA; ^6^ Charité ‐ Universitätsmedizin Berlin Germany

**Keywords:** memory, PIRCHE, SLA, swine, xenotransplantation

## Abstract

Swine‐derived kidneys are a promising alternative organ source for transplantation, but compatibility in the major histocompatibility complex remains an immunological barrier. Furthermore, in repeat transplantations, CD4+ memory T cells can lead to a more rapid immune response against repeated exposure to the same antigens. Several studies have shown that HLA and SLA proteins share overlapping B cell epitopes due to structural or electrostatic similarities, but the role of overlapping T cell epitopes has not been fully explored. This study aims to computationally analyse the potential risk of memory T cell activation in subsequent human‐after‐swine and swine‐after‐human transplantation by evaluating shared T cell epitopes between the two graft sources. We show that while HLA and SLA demonstrate striking structural similarities, their linear protein sequences are very distinct, which translates to disparate HLA‐ and SLA‐derived peptidomes and T cell epitopes. By applying the PIRCHE‐II Tmem analysis to a simulated panel of recipients receiving repeat transplantations from a human kidney and from a swine xenograft, we observed a median of 1 shared T cell epitope in the cross‐species context, compared to a median of 17 shared between two human‐derived kidneys. This suggests that a swine xenograft exposes a low risk of T cell memory against a later human donor, and that xenotransplantation may provide an opportunity to receive a graft for highly HLA‐sensitised recipients.

**Abbreviations**
MHC: Major Histocompatibility Complex
PDB: Protein Data Bank
PIRCHE: Predicted Indirectly Recognisable HLA Epitopes
RCSB: Research Collaboratory for Structural Bioinformatics
RMSD: Root Mean Square Deviation
SLA: Swine Leukocyte Antigen
SOT: Solid Organ Transplantation
STEP: Shared T‐cell EPitopes

## 1 Introduction

Kidney transplantation is an effective treatment for renal failure. However, mismatched antigens between recipient and donor can provoke immune responses and lead to adverse transplant outcomes. Evaluating HLA antigens of the recipients and donors and detecting pre‐existing donor‐specific HLA antibodies in the recipient can enhance compatibility and improve transplant outcomes [1, 2, 3].

Due to limited availability of human kidneys, swine‐derived kidneys are being developed as an alternative organ source for transplantation [4], highlighted by the recent successes in Pig‐to‐Human kidney transplantation [5]. Similar progress has also been made in heart xenotransplantation [6, 7] and in swine liver transplantation [8]. Despite these developments, significant challenges remain [9]. A major barrier is the presence of human antibodies against the swine galactose α‐1,3 galactose (αGal) antigen, which can trigger hyper‐acute rejection of grafts expressing this antigen. To address this issue, swine have been bioengineered to knock out multiple genes related to xenoantigens, including αGal. This genetic engineering has significantly enhanced the feasibility and success of xenotransplantation [10, 11].

**FIGURE 1:**
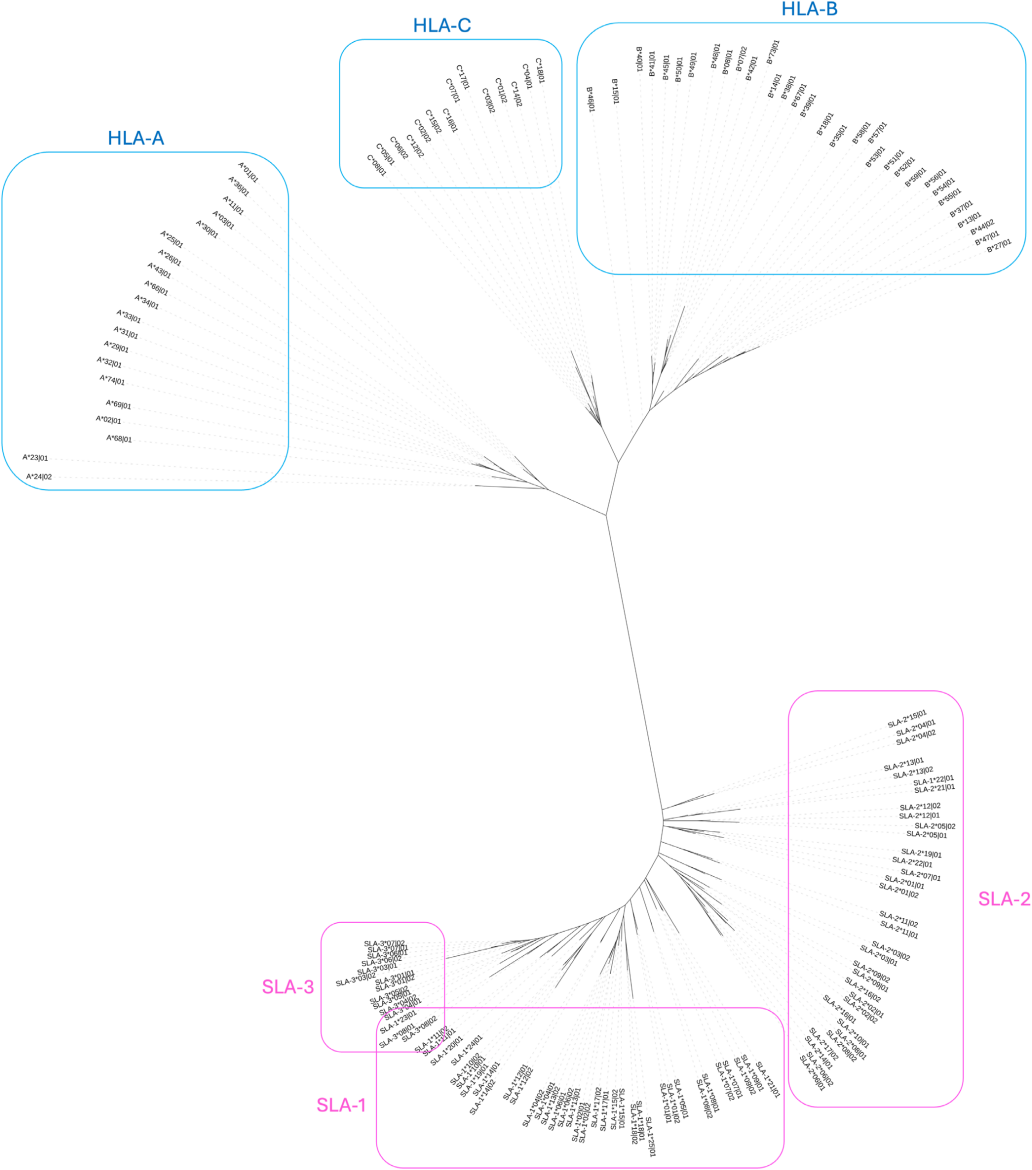
Phylogenetic tree of representative HLA and SLA protein sequences. Phylogenetic trees show comparative similarity of representative MHC Class I amino acid sequences. All HLA‐A, ‐B and ‐C sequences (blue boxes and labels) are more similar to each other than any of the SLA sequences, and all SLA ‐1, ‐2, and ‐3 (pink boxes and labels) sequences are more similar to each other than any of the HLA sequences. Phylogenetic trees of select MHC Class II Alpha chain and Beta chain are shown in Figures S1A and S1B, respectively.

Despite advancements in bioengineered knockout swine, significant immunological risks remain in xenotransplantation [12, 13], particularly regarding compatibility in the major histocompatibility complex (MHC), which remains an immunological barrier [14]. The HLA [15] and Swine Leukocyte Antigen (SLA) [16, 17, 18] genes encode highly polymorphic cell‐surface proteins (Figure 1). Analogous to humans, the swine MHC contains clusters of classical and non‐classical MHC genes in the Class I region. However, orthology between the respective MHC Class I genes of humans and swine has not been specifically defined [17].

Several studies have indicated that HLA and SLA proteins might share overlapping B cell epitopes, and human anti‐HLA antibodies may be cross‐reactive towards SLA [10, 19, 20, 21, 22, 23, 24]. Recent computational analyses identified and quantified solvent‐accessible shared amino acids shared between non‐human primates and swine [25]. These findings support the hypothesis that antibody cross‐reactivity is driven by the presence of structurally similar, antibody‐accessible amino acids. As a result, patients with pre‐existing anti‐HLA antibodies may generate rapid immune responses against Swine xenotransplants. These immune responses may be partially alleviated by specific immunosuppression treatments [26], or by downregulating MHC class I expression in the graft [27, 28]. Moreover, strategies involving precise molecular matching may help to identify immunological gaps and improve graft compatibility.

The PIRCHE‐II algorithm is a molecular matching algorithm for predicting and comparing HLA‐derived T cell epitopes as a risk assessment in transplantation [29]. PIRCHE‐II predicts a set of donor‐MHC‐derived peptides which are presented by a recipient's HLA Class II (HLA‐DR) molecules to CD4+ T cells. The resulting PIRCHE‐T2 score corresponds to the number of potential helper T cell epitope targets, which reflects a degree of immunological risk. Elevated PIRCHE‐T2 scores have been shown in numerous studies to correlate with transplantation risk and outcomes [30, 31, 32]. The PIRCHE algorithms have also recently added capabilities to analyse B cell epitopes [33, 34] (PIRCHE‐B), to predict potential targets of antibody reactivity following the concept of linked recognition [35, 36].

In repeat transplantations, immunological memory can lead to a more rapid immune response against repeat antigens [37]. Prior exposure to donor MHC molecules can induce B cell and T cell memory, allowing for a more rapid, stronger, or sustained response to a re‐exposure to the same antigen. Assessing epitope overlap between sequential transplants gives indications of a potential memory‐driven immune response and the risk of immunological events. The PIRCHE‐Tmem module and corresponding Shared T‐cell EPitopes (STEP) score can give indications that there can be distinguishable differences in risk based on repeat exposure [38, 39]. Although originally developed to assess risk in repeat human transplantation, the STEP concept may also be applied to evaluate the potential for anti‐HLA memory T cells to cross‐react with swine antigens in the case of xenotransplants. Such an approach could further support risk stratification and help guide donor selection in xenotransplantation settings.

To our knowledge, no major studies have structurally explored the role of cross‐reactive T cell epitopes between humans and swine MHC. This study aims to computationally analyse the compatibility of swine xenotransplants by prediction of potential SLA‐derived CD4+ T cell epitopes presentable by recipient HLA. Using a recently integrated module within the TxPredictor platform, we predicted HLA‐DRB1‐presented epitopes from swine donors and compared these predictions against epitopes from human transplantations as a relative risk comparison. Additionally, we explore the potential risk of memory T cell activation in subsequent human‐after‐swine and swine‐after‐human transplantation by computationally evaluating shared epitopes from the two graft sources.

## 2 Materials and Methods

### 2.1 Sequence Analysis: HLA and SLA

To understand the sequence differences between HLA and SLA, we compared MHC sequences between the two species. To this end, full‐length protein sequences of HLA‐A, ‐B, ‐C, ‐DRB1, ‐DRB3, ‐DRB4, ‐DRB5, ‐DRA, ‐DQB1, and ‐DQA1 were extracted from the IPD‐IMGT/HLA database (v 3.57.0) [15], as well as swine SLA‐1, ‐2, and ‐3, ‐DRB1, ‐DRA, ‐DQB1, ‐DQA from the IPD‐MHC database [40]. The sequence dissimilarity between pairwise sequences is quantified using a Hamming distance, which is calculated as a count of the number of amino acid positions that differ between the two. We aligned protein sequences using MUSCLE (v 5.3) [41] and generated phylogenetic trees using Phyml (v 3.1) [42]. We used these phylogenetic trees to explore the relationship between HLA and SLA linear protein sequences.

To analyse differences in potential T cell epitope targets, we performed analysis on the unique peptidomes within HLA and SLA sequences. All full‐length provided HLA protein sequences within the IPD‐IMGT/HLA database (v 3.57.0) were selected, and all SLA protein sequences within the IPD‐MHC database were extracted. To identify potential T cell epitope targets, we used a sliding window to list all possible 15mer sequences within the MHC sequences. We analyse which of these unique peptides are shared or unique between the human and swine MHCs. These 15mer peptides are used in downstream analysis in the molecular matching analysis.

For the purpose of this study, and to facilitate analysis of swine xenotransplantation by the HLA community, SLA sequences were recently enabled in the TxPredictor package for molecular matching (PIRCHE‐T2) and T cell memory (PIRCHE‐Tmem) analysis. Peptide binding analysis was performed for all swine‐derived peptides, resulting in a peptide binding rank for each peptide in the context of the binding cleft of human HLA‐DR molecules. This binding prediction is the same as the previously presented process for human‐derived peptides [36], and gives a relative binding score for predicted swine‐derived T cell epitopes. Swine MHC molecules are not analysed for their capabilities in peptide presentation.

### 2.2 Structure Analysis

To focus on structural changes, we selected representative protein structures from the Research Collaboratory for Structural Bioinformatics (RCSB) Protein Databank (PDB) database (https://www.rcsb.org/ [43]). The PDB database was searched for humans and swine MHC class I structures using the keywords “HLA”, “HLA‐A”, “HLA‐B”, “HLA‐C”, “SLA”, “SLA‐1”, “SLA‐2”, “SLA‐3” to identify candidate MHC structures. For the candidate protein structures, the amino acid sequences contained within structure chains named “A”, “B”, and “C”, which usually correspond to the MHC alpha chain, the beta 2 microglobulin, and the peptide chains, respectively, were extracted for further analyses. In parallel, the amino acid sequences were compared using BLAST [44] against a database of MHC Class I and II sequences from the IPD‐IMGT/HLA (v 3.57.0) and IPD/MHC databases. BLAST hits with the highest number of identical amino acids were used to determine which specific MHC sequence is present within the structure. Up to three representative structures with a maximum BLAST alignment score per MHC 2‐field allele name [45, 46] were identified and used in subsequent pairwise structure comparisons.

Representative protein structures were compared to each other for their shape and folding using the FATCAT “rigid” algorithm [47] as provided in the PDB web interface. This algorithm aligns structures by minimising the Root Mean Square Deviation (RMSD), measured in Angstroms, which estimates the mean distance between corresponding atoms in two structures. The algorithm provides a similarity score, which is a unitless measure of structural similarity based on the RMSD, the length of the aligned amino acid sequence, and secondary structure features of the aligned proteins. For our analysis, all structure comparisons were stratified based on the relationship of the source MHC molecules, that is, identical structures, within and across gene groups, and across species.

### 2.3 Memory Analysis

Four thousand virtual patients and 1000 donors were randomly generated as previously described [48] using weighted 2007 NMDP haplotype frequencies [49], and paired to simulate a cohort of 4 million randomly allocated transplantations. The same 4000 virtual patients were also paired with a well‐characterised reference SLA genotype which is in common use for xenotransplant studies (SLA‐1*07:02 + SLA‐1*13:01 + SLA‐1*12:01^SLA‐2*10:01 + SLA‐2*02:02^SLA‐3*05:02 + SLA‐3*04:02^SLA‐DRB1*10:01 + SLA‐DRB1*04:03^SLA‐DQB1*06:01 + SLA‐DQB1*03:03^SLA‐DQA*02:04 + SLA‐DQA*01:01^SLA‐DRA*02:01 + SLA‐DRA*04:01) [50, 51]. Potential donor‐derived T cell epitopes which are likely presented by patient HLA‐DRB1 were predicted using the TxPredictor web service to give a baseline PIRCHE‐T2 compatibility score distribution. These score distributions were calculated for the randomly paired human donors, as well as the reference swine donor, and distributions from human transplantation and xenotransplantation were compared.

Potential T cell memory (Tmem) induced by HLA or SLA immunisation was evaluated using the TxPredictor Tmem module. This algorithm predicts HLA‐DRB1‐presented epitopes derived from two separate donor genotypes, for example between the randomly paired human donor and the swine donor. Epitopes derived from the second graft which overlap(match) with those derived from the first graft are counted and considered as T cell memory risk score. The PIRCHE‐Tmem scores were calculated in both the swine‐after‐human (SAH) and HAS context to compare the “directionality” of shared epitopes in subsequent transplantations.

## 3 Results

### 3.1 Sequence Comparisons

A phylogenetic tree of representative MHC proteins is shown in Figure 1. HLA and SLA variants form completely separate clades, indicating that all Class I HLA protein sequences are more similar to other HLA than to any of the Class I SLA. Likewise, the SLA proteins cluster together, showing higher similarity among themselves than with any HLA sequence. Average Hamming distance between MHC sequences is shown in Table 1. On average, HLA and SLA class I sequences differ by 196.48 amino acids.

**TABLE 1:** Mean structural similarity and sequence divergence of SLA and HLA class I molecules.

	*N*	Mean structural similarity	Mean RMSD	Mean hamming distance
Same PDB ID	138	816.0	0.0	0.0
Same Allele ID, different structure	204	814.0	0.55	0.0
Same locus, same species	6996	813.3	0.68	42.84
Different locus, same species	8912	808.5	0.97	105.67
Different species	2794	806.2	1.15	196.48

*Note:* Comparisons of select HLA and SLA structures and their corresponding linear amino acid sequences are shown. “Mean Structural Similarity” and “Mean RMSD” are algebraic means of the similarity scores and RMSD values for all FATCAT rigid structure alignments. “Mean Hamming Distance” is calculated based on a count of differing amino acids in pairwise comparisons of all aligned amino acid sequences and represents a measure of sequence dissimilarity. HLA and SLA structures are quite similar based on structural similarity scores, but the Hamming distance suggests that linear amino acid sequences of HLA and SLA are very different.

From the available HLA amino acid sequences (HLA‐A, ‐B, ‐C, ‐DRA, ‐DRB1, ‐DRB3, ‐DRB4, ‐DRB5, ‐DQA1, ‐DQB1, ‐DPA1, ‐DPB1) within IPD‐IMGT/HLA, we identified 158,083 unique 15mer sequences. Similarly, from the available SLA sequences (SLA‐1, ‐2, ‐3, ‐DRA, ‐DRB1, ‐DRB2, ‐DRB3, ‐DRB4, ‐DRB5, ‐DQA, ‐DQB1), we identified 13,969 unique 15mers, which reflect the smaller number of available MHC sequences. From the HLA‐ and SLA‐derived peptidomes, only 894 (0.5% of the combined HLA and SLA peptidomes) of the unique 15mer sequences are present in both pools, indicating a small overlap in the cross‐species MHC‐derived peptidomes. These unique 15mers, when paired with a corresponding presenter HLA molecule, represent the potential T cell targets used in molecular matching and T cell memory analysis.

### 3.2 Structure Comparisons

**FIGURE 2:**
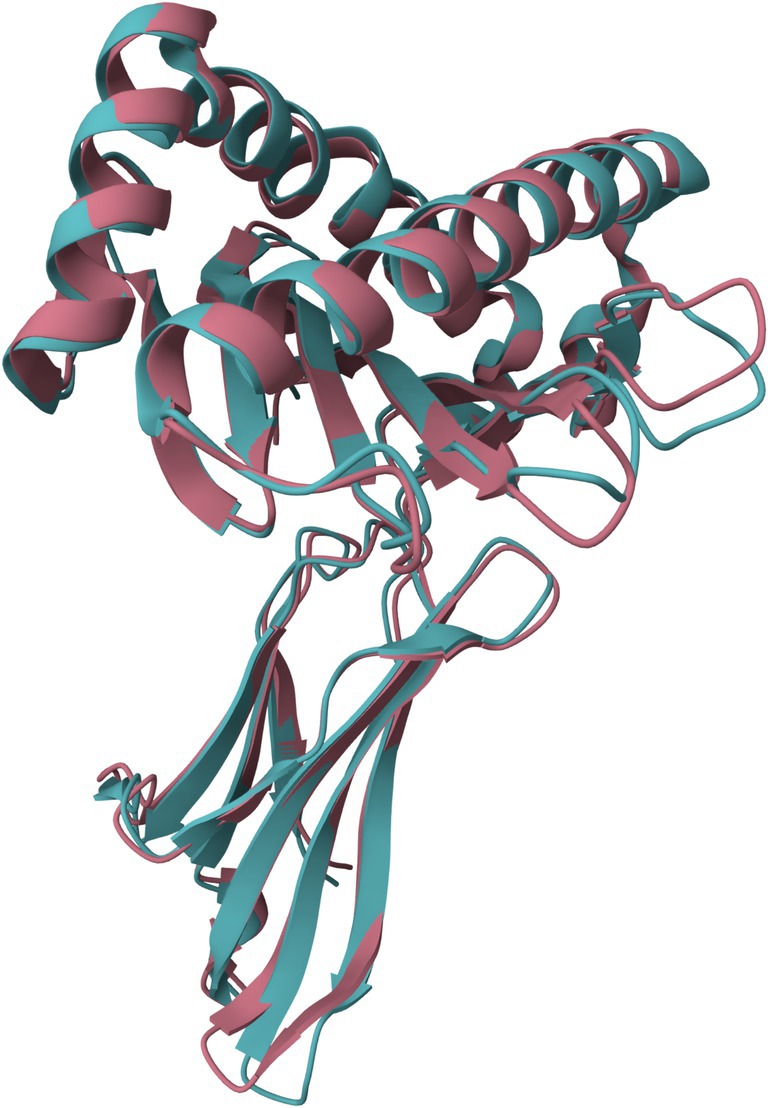
A representative alignment of an HLA and SLA protein structure. A FATCAT alignment of PDB structures 7EMA (PDB DOI: 10.2210/7ema/pdb, SLA‐1*01:01) and 1QVO (PDB DOI: 10.2210/1qvO/pdb, HLA‐A*11:01) is shown. The protein structures mostly overlap, indicating a high degree of structural homology.

Using the defined search keywords, we identified 868 unique protein structures. After mapping which specific HLA or SLA Class I allele is present in the identified PDB structures, up to three structures for each available allele were selected for closer comparison. This resulted in a total of 138 protein structures representing 66 distinct proteins (7 SLA and 59 HLA). Each of these 138 protein structures was aligned against all other structures using the FATCAT rigid alignments, resulting in 19,044 comparisons. A representative structure alignment is shown in Figure 2, which shows an SLA‐1*01:01 structure overlapping with a structure containing HLA‐A*11:01.

Each alignment generates a structural similarity score and Root Mean Square Deviation (RMSD), which are estimations of similarity (or dissimilarity) of two structures respectively. The structural similarity scores, stratified by matching context, are shown in Table 1. We observe a mean structural similarity of 816.0 when structures are aligned against themselves, indicating an “upper bound” of expected values. As the MHC sequences become gradually less similar (i.e., structures containing the identical MHC sequence, within the same gene, within the same species) we observed the mean structural similarity correspondingly becoming lower, with a cross‐species mean of 806.2. Likewise, the RMSD scores become correspondingly higher in cross‐species structure analysis (mean = 1.15).

**FIGURE 3:**
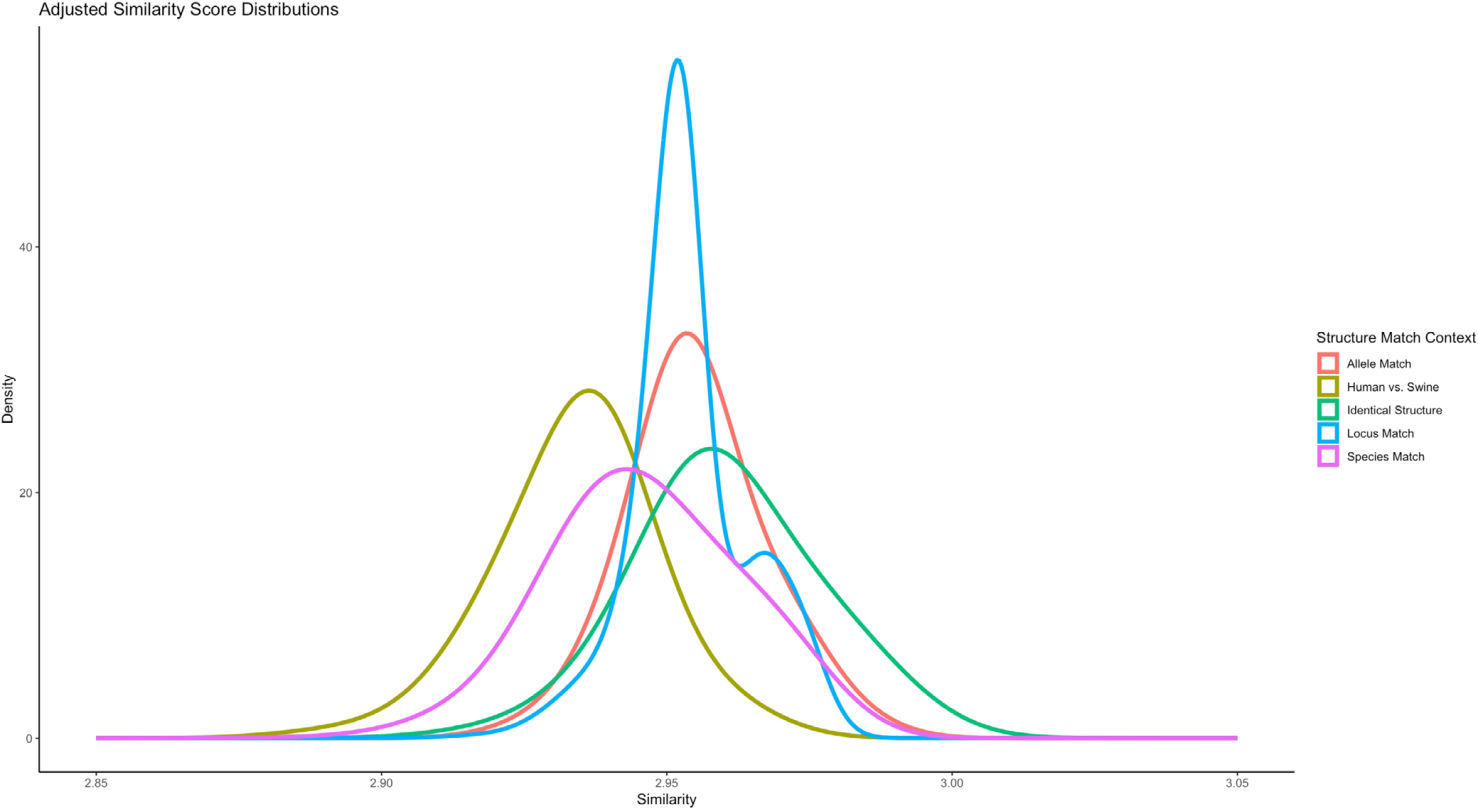
Structural MHC similarity for various matching contexts. The “Adjusted Similarity Score” (SS divided by aligned sequence length) from pairwise structure alignments. Density curves are stratified based on if the two crystal structures are the exact same PDB crystal structure (green), derived from the same MHC protein (red), from the same human or swine MHC locus (blue), separate loci from the same species (pink) or are from separate species (yellow). While more similar MHC molecules expectedly show more similar protein structures, in all cases the comparisons fall within overlapping range.

Since structural similarity scores were observed to be dependent on the aligned sequence length, for subsequent comparisons structural similarity was adjusted by dividing by the length of the aligned amino acid sequences. In Figure 3 we see a density distribution of adjusted structural similarity stratified by structure/allele/locus/species matching contexts. When a protein structure is compared against itself (green), we observe comparatively high adjusted structural similarity (Table 1). The distribution showing structures originating from the same allele (red) is nearly as similar as comparisons of identical structures. These score distributions are slightly lower when comparing structures with MHC proteins derived from different alleles, loci, or species (blue, pink, yellow, respectively). Interestingly, all of these distributions of scores are heavily overlapping, indicating that, even when comparing cross‐species MHC structures, all of these comparisons fall into the same general range.

**FIGURE 4:**
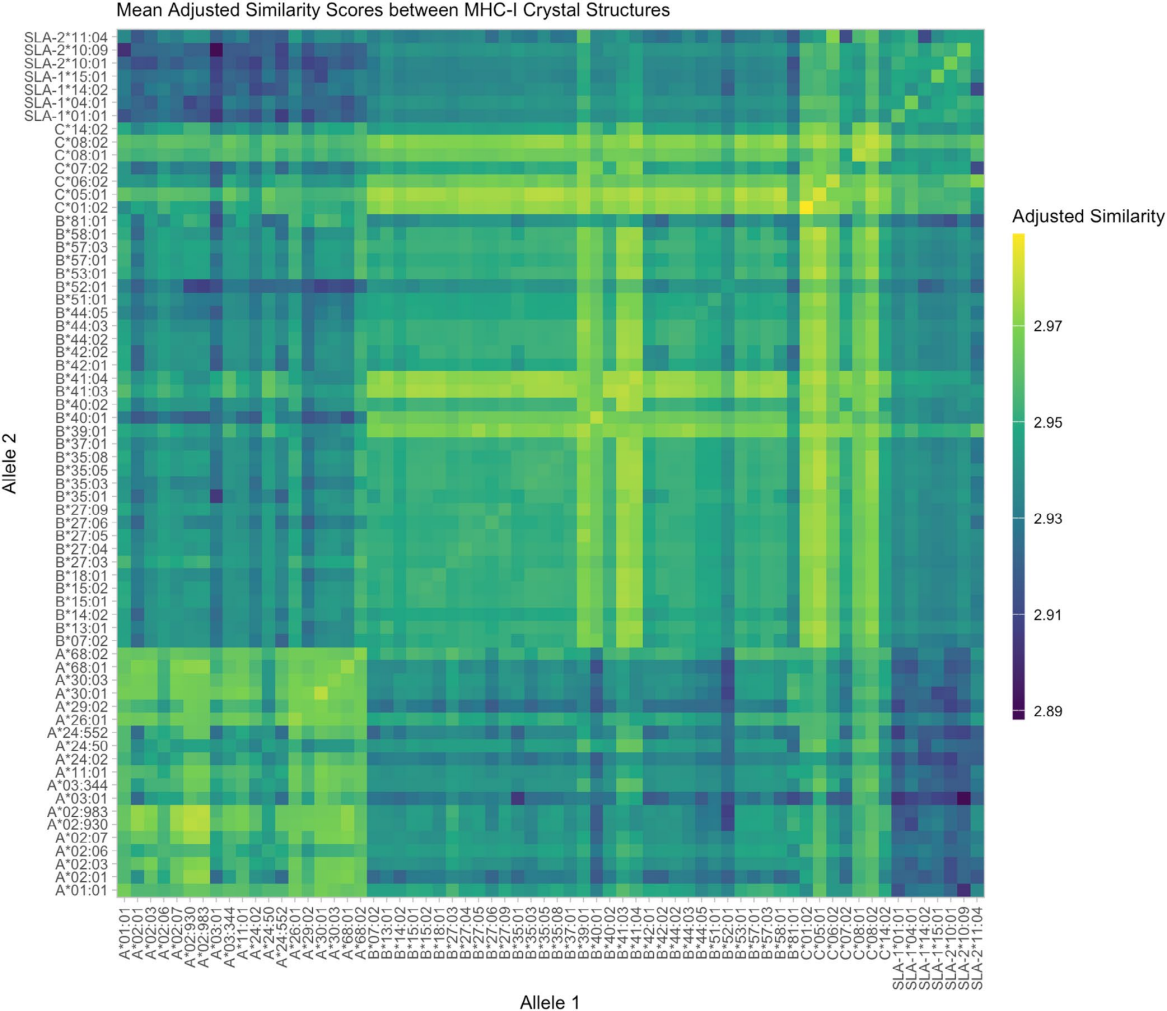
Heatmap of average adjusted structural similarity per MHC protein. Mean adjusted structural similarity scores from all structure alignments are stratified by the source MHC allele. We observe generally higher similarity for identical structures (diagonal) and for comparisons of alleles from within the same locus, with lower structural similarity for comparisons across species (darker blue).

Similarity scores adjusted by aligned sequence length and stratified by source MHC alleles were plotted into a heatmap (Figure 4). Several general trends are observed, which reflect our previous observations of structural similarity. We observe generally higher similarity for identical structures (diagonal), and for comparisons of alleles from within the same locus. Comparisons of structures from different loci or species are comparatively lower similarity, but in all cases the structures are very similar, indicating a conserved MHC Class I structure.

### 3.3 PIRCHE Analysis

**FIGURE 5:**
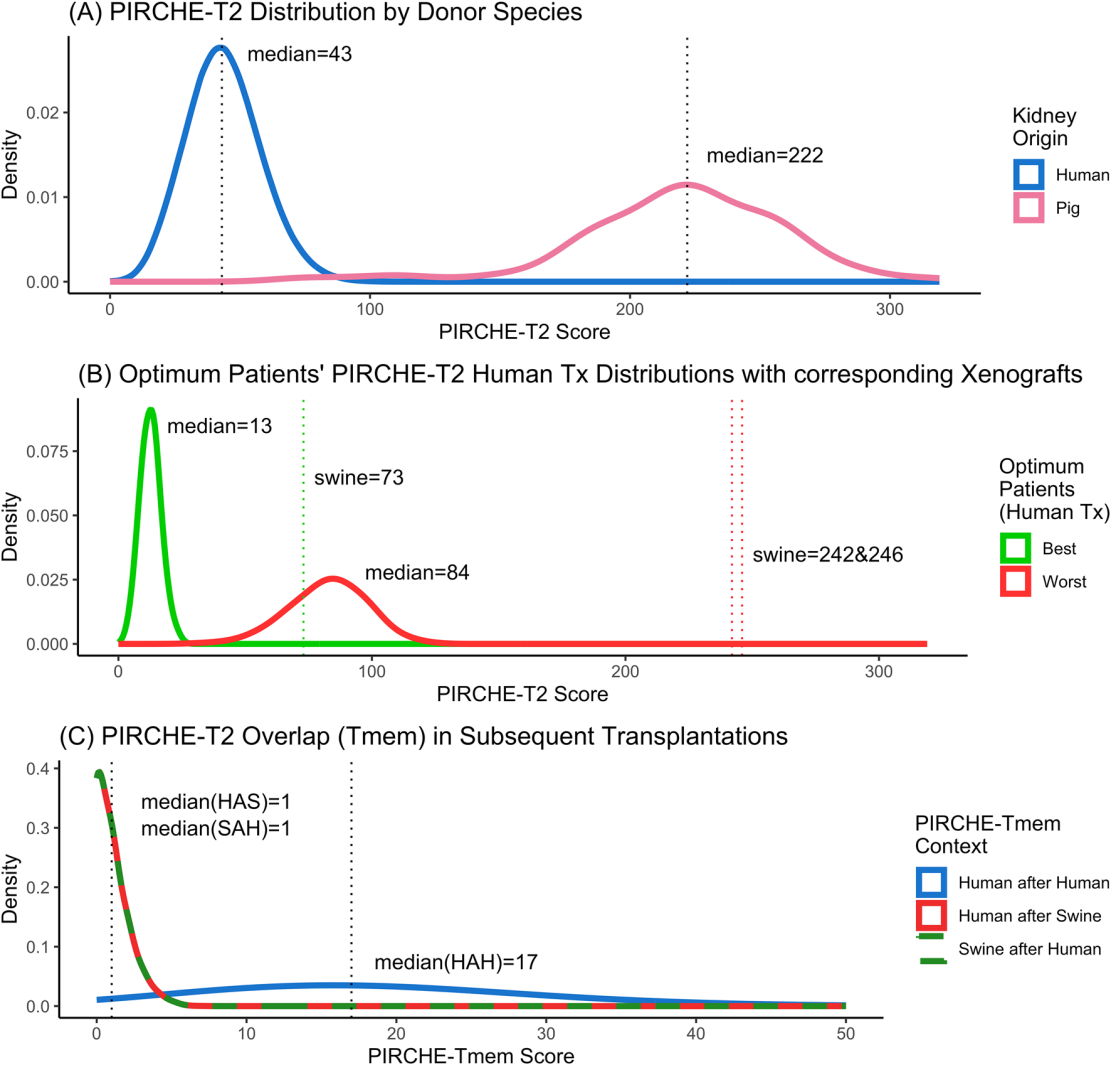
PIRCHE‐T2 and PIRCHE‐Tmem score density for human‐ & swine‐derived transplantations. (A) Distributions of PIRCHE‐T2 risk scores for the two kidney origin species. Distributions from swine‐derived transplants demonstrate a higher predicted risk than human‐derived transplants. (B) Distributions of PIRCHE‐T2 risk scores across the simulated donor pool for the optimum (lowest and highest median scores) patients. One patient was found with the lowest median PIRCHE‐T2 score (13) and two patients were identified with the same worst‐case median PIRCHE‐T2 score (84). These best‐ and worst‐case patients' corresponding scores from swine‐derived grafts (73, and 242&246, respectively) are shown as dotted lines. The swine‐derived transplant has a much higher predicted risk than all human‐derived grafts for all patients in our simulated panel, exemplified in the optimum patients. (C) Distributions of PIRCHE‐Tmem scores for repeat transplantations. PIRCHE‐Tmem scores are higher in two subsequent human grafts, compared with cross‐species grafts.

Swine xenotransplantations demonstrated significantly higher PIRCHE‐T2 scores compared with randomly paired human transplants (Figure 5A). From within the human‐derived distribution, we selected the simulated patients with the lowest (best‐case, green, median = 13) and highest (2x, worst‐case, red, median = 84) median PIRCHE‐T2 scores across the donor population for further analysis (Figure 5B). These simulated patients represent individuals for whom we would expect to easily find a well‐matching donor, or with a low chance of finding a well‐matched donor, respectively. We can see that in the best‐case patient as well as the worst‐case patients, the reference swine xenograft indicated much higher PIRCHE‐T2 scores than their respective distribution of human‐derived grafts (Figure 5B). In every patient, we observed that the reference swine xenograft has a much higher PIRCHE‐T2 score than any of the randomly assigned human donors.

Analysis of the “directionality” (order) of subsequent transplants revealed that PIRCHE‐Tmem scores for SAH (Figure 5C, green) and HAS (Figure 5C, red) transplants were identical, indicating no difference in the order of transplants. Distributions of Tmem scores of both SAH and HAS are notably lower than a human‐after‐human (HAH) control group (Figure 5C, blue), with 44.9% of the SAH or HAS patients sharing zero T cell epitopes. In contrast, HAH memory scores showed a median shared epitope score of 17, indicating a higher predicted T cell memory risk than SAH and HAS (median = 1).

## 4 Discussion

In this study, we explored shared xenograft‐derived T cell epitopes using an extensive computational approach. To the best of our knowledge, our report describes the first study that structurally addresses the potential of human T cell responses towards swine‐derived MHC upon transplantation. Despite the structural similarities between SLA and HLA, our observations of the dissimilarity in amino acid sequences indicated an expectation of large differences in SLA‐ and HLA‐derived linear epitopes. While these differences could lead to a large CD4+ T cell response in a swine xenograft, the SLA‐ and HLA‐derived epitope repertoires showed only minor overlap, which may be advantageous in subsequent swine after human or human after swine transplantations.

The high structural similarity between all MHC class I molecules regardless of species was striking. By comparing exactly identical structures, we observe an “upper bound” of structural similarity scores which represent a “good” alignment, as seen in Figures 3 and 4. Even when comparing cross‐species structures, the distribution of similarity scores heavily overlaps distributions comparing identical structures. Since antibodies are often defined based on specific structures on the surface of HLA molecules [25, 34, 52], this similarity likely contributes to why anti‐HLA antibodies can be cross‐reactive against SLA molecules.

In contrast to the structural similarity, the sequence comparisons shown in the phylogenetic tree in Figure 1 showed that the HLA Class I sequences are distinct from the SLA Class I. As they are derived from separate species, this observation is unsurprising. It does however indicate that there is value in looking closely at the specific differences and their role in defining differences in derived peptidomes, which plays a role in defining the MHC molecules' immunogenicity.

By analysing MHC‐derived peptidomes, we obtain a view of the potential diversity of helper T cell epitopes (defined as donor‐MHC‐derived peptides presented in a patient's HLA Class II) to which a patient is exposed in transplantation. The unique peptidome count for human‐derived (158,083) 15mer peptides is notably higher than the count of swine‐derived (13,969) 15mers, which can be attributed to a number of reasons. Human MHC is more widely studied than swine MHC, and therefore more HLA sequences are available due to their need in transplantation registries. Additionally, swine are artificially selected for breeding, and this is likely to be especially true when selecting specific pigs for use in transplantation, which may further limit their MHC polymorphism. It is interesting that between the human and swine peptidomes, only 894 of these peptides are present in both datasets. This observation drives the hypothesis that we expect very few shared T cell epitopes between human and swine grafts.

The higher PIRCHE‐T2 scores predicted in xenotransplants show that swine donors provide higher loads of MHC‐derived T‐helper cell epitopes compared to human donors in all cases. This finding is not surprising, due to the evolutionary divergence and distinct nucleotide and amino acid sequences between HLA and SLA. This suggests that when possible, nearly any healthy human‐derived graft is likely preferable to a swine‐derived graft. Since epitope scores will always be high in swine xenotransplantation, this indicates a potential limitation on the utility of molecular mismatch load analysis as a risk assessment tool, since all patients might be considered high‐risk compared to human donors.

Molecular matching has an important role in predicting epitopes present in both sequential (e.g., human‐ and swine‐ derived) grafts. By counting the shared epitopes, we can quantify a predicted risk of T cell memory caused by an immunizer epitope in the initial exposure, which may lead to a memory response in subsequent grafts. In the case of two cross‐species grafts, we observe very few shared epitopes compared with two human‐derived grafts. Scores were identical regardless of the order of transplantation (human‐after‐swine or swine‐after‐human).

The lack of shared epitopes suggests that receiving an initial xenograft exposes a low risk of developing anti‐HLA‐specific T cell memory, which might be reactivated upon exposure to a later human donor. In addition, it also suggests that an initial human graft, which is likely to lead to de novo anti‐HLA antibodies, might introduce low immunologic risk of T cell memory on a subsequent swine xenograft. In particular, for highly HLA‐sensitised recipients with panel‐reactive antibody levels close to 100%, xenotransplantation may provide an opportunity to receive a graft with manageable immunological risk.

The observation that swine‐derived grafts would lead to very low predicted T cell memory initially seems to contradict the observation that swine‐derived grafts lead to very high PIRCHE‐T2 scores. The low memory however can be explained by the very distinct peptidomes derived from swine and human MHC. The lack of predicted memory is a direct result of the near absence of shared swine‐ and human‐MHC‐derived peptides. In contrast, in the setting of two subsequent human‐derived grafts, we are much more likely to find shared MHC‐derived peptides between the grafts, and therefore observe comparatively higher PIRCHE‐Tmem scores.

The low predicted shared T cell memory contrasts observations in previous studies which identified cross‐reactivity of anti‐HLA antibodies and SLA. As suggested previously [20, 24], this antibody cross‐reactivity is likely caused in part by structural or electrostatic similarities between epitopes on these molecules, a conclusion which is reinforced by our observed similarities between HLA and SLA structures. (Figures 2 and 3) Further experiments are warranted to determine if cross‐reactivity can be further explained by models that predict the accessibility of shared amino acids between these structures [33, 34], or to see if gaps in antibody cross‐reactivity could be explained by comparably limited overlap in MHC‐derived T cell epitopes.

By understanding which immune process leads to adverse responses, it may help in directing interventions. By thoroughly exploring the role of swine‐derived epitopes in their role in immune response and development of memory, we may give context to immune targets which might be genetically modified or downregulated. Efforts can be focused on compatibility of problematic B cell epitopes to more accurately assess histocompatibility. Additionally, some studies have suggested that using point mutations may reduce cross reactivity for antibodies [20]. In the rare cases where there may be shared predicted memory epitopes, perhaps these point mutations to alter the expressed SLA‐derived peptidome in the swine graft may further reduce the risk of shared epitopes.

Analysis of molecular structures in this study is focused on MHC Class I. This is a practical limitation; to our knowledge, there are very few SLA Class II structures available in the PDB database. Furthermore, T cell memory analysis is focused on epitopes as defined by the PIRCHE‐T2 algorithm, which is a specific HLA Class II presenter with its specific 9mer core peptide. It is possible that non‐identical but similar peptides may have cross‐reactive T cell responses, and a more lenient definition of a unique epitope could lead to higher shared epitope scores.

Genetically modified pig kidneys remain an exciting opportunity and a promising source of organs for patients. By adapting the PIRCHE‐II algorithm to xenotransplantation, we estimate generally higher molecular mismatch loads derived from swine grafts compared to human allografts. The predicted limited overlap between HLA‐derived T cell epitopes with SLA‐derived epitopes may represent an opportunity for highly HLA‐sensitised recipients with little to no chances of finding an organ donor.

## Conflicts of Interest

The UMC Utrecht has filed patent applications on the prediction of an alloimmune response against mismatched HLA. E.S. is listed as an inventor on these patents. B.M.M. and M.N. are employed by PIRCHE AG, which publishes the PIRCHE web portal.

## Supporting information


**Figure S1A.** Phylogenetic tree of representative HLA and SLA class II alpha chain amino acid sequences. Similar to the MHC class I sequences shown in Figure 1, clades containing human‐ and swine‐derived sequences are distinct from each other. Interestingly, the HLA‐DRA and SLA‐DRA sequences appear more similar than comparisons of different loci within the same species (e.g., HLA‐DQA1 against HLA‐DRA).
**Figure S1B.** Phylogenetic tree of representative HLA and SLA class II beta chain amino acid sequences. Similar to the MHC class I sequences shown in Figure 1, clades containing human‐ and swine‐derived sequences are distinct from each other. Similar to the alpha chain sequences, the HLA‐DRB1 and SLA‐DRB sequences appear more similar than comparisons of HLA‐DRB1 against HLA‐DQB1.

## Data Availability

The data that support the findings of this study are available from the corresponding author upon reasonable request.
