# Supplementary material for: Swine Xenografts Share Few Predicted Indirectly Recognisable SLA‐Derived Epitopes With HLA‐Derived Epitopes From Human Kidney Grafts
Source: HLA. 2025 Jun 21;105(6):e70291. doi: 10.1111/tan.70291 (PMC12182435; doi:10.1111/tan.70291)
Supplement: Supplementary file 1 — Figure S1A. Phylogenetic tree of representative HLA and SLA class II alpha chain amino acid sequences. Similar to the MHC class I sequences shown in Figure 1, clades containing human‐ and swine‐derived sequences are distinct from each other. Interestingly, the HLA‐DRA and SLA‐DRA sequences appear more similar than comparisons of different loci within the same species (e.g., HLA‐DQA1 against HLA‐DRA). Figure S1B. Phylogenetic tree of representative HLA and SLA class II beta chain amino acid sequences. Similar to the MHC class I sequences shown in Figure 1, clades containing human‐ and swine‐derived sequences are distinct from each other. Similar to the alpha chain sequences, the HLA‐DRB1 and SLA‐DRB sequences appear more similar than comparisons of HLA‐DRB1 against HLA‐DQB1. [file TAN-105-e70291-s001.docx]

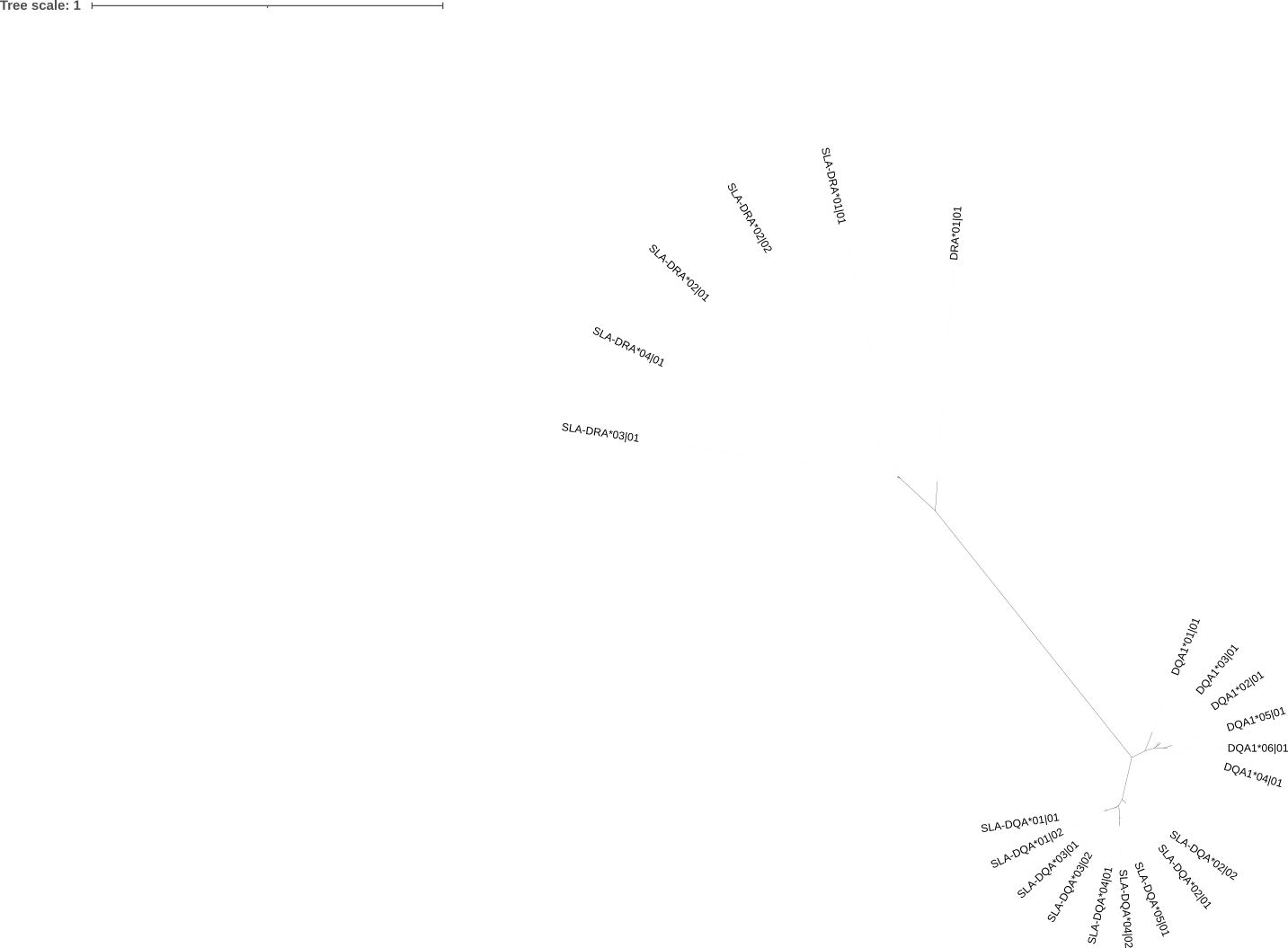


**Supplementary Fig. 1A. Phylogenetic tree of representative HLA and SLA class II alpha chain amino acid sequences.**

Similar to the MHC class I sequences shown in Figure 1, clades containing human- and swine-derived sequences are distinct from each other. Interestingly, the HLA-DRA and SLA-DRA sequences appear more similar than comparisons of different loci within the same species (*e.g.* HLA-DQA1 against HLA-DRA).


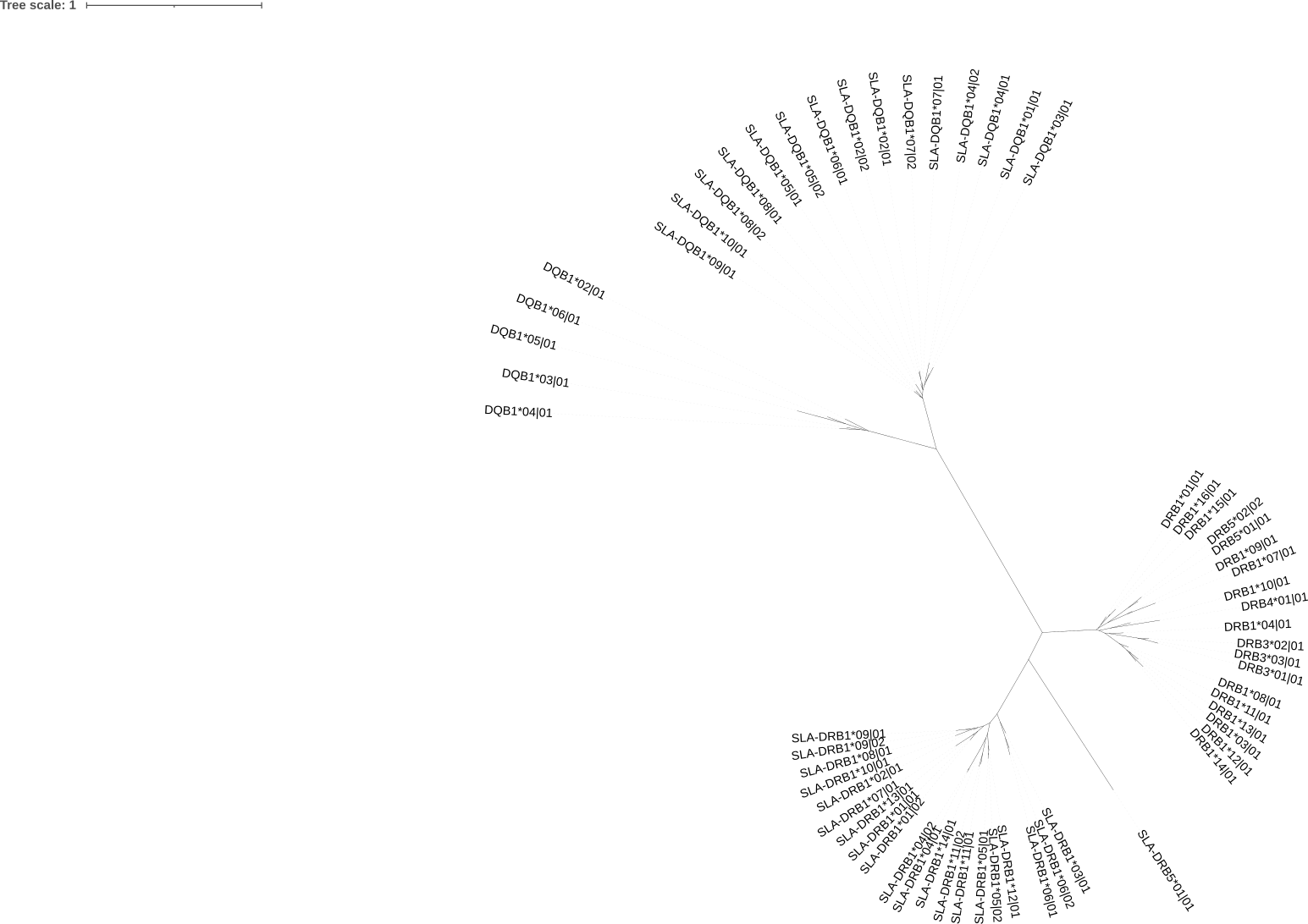


**Supplementary Fig. 1B. Phylogenetic tree of representative HLA and SLA class II beta chain amino acid sequences.**

Similar to the MHC class I sequences shown in Figure 1, clades containing human- and swine-derived sequences are distinct from each other. Similar to the alpha chain sequences, the HLA-DRB1 and SLA-DRB sequences appear more similar than comparisons of HLA-DRB1 against HLA-DQB1.
